# Hyaluronan-CD44 interactions mediate contractility and migration in periodontal ligament cells

**DOI:** 10.1080/19336918.2019.1568140

**Published:** 2019-02-08

**Authors:** Zeinab Al-Rekabi, Adriane M. Fura, Ilsa Juhlin, Alaa Yassin, Tracy E. Popowics, Nathan J. Sniadecki

**Affiliations:** a Department of Mechanical Engineering, University of Washington, Seattle, WA, USA; b Department of Bioengineering, University of Washington, Seattle, WA, USA; c Department of Periodontics, University of Washington, Seattle, WA, USA; d Department of Oral Health Sciences, University of Washington, Seattle, WA, USA; e Institute for Stem Cell and Regenerative Medicine, University of Washington, Seattle, WA, USA

**Keywords:** Traction forces, cell migration, hyaluronan, CD44, periodontal ligament cells

## Abstract

The role of hyaluronan (HA) in periodontal healing has been speculated via its interaction with the CD44 receptor. While HA-CD44 interactions have previously been implicated in numerous cell types; effect and mechanism of exogenous HA on periodontal ligament (PDL) cells is less clear. Herein, we examine the effect of exogenous HA on contractility and migration in human and murine PDL cells using arrays of microposts and time-lapse microscopy. Our findings observed HA-treated human PDL cells as more contractile and less migratory than untreated cells. Moreover, the effect of HA on contractility and focal adhesion area was abrogated when PDL cells were treated with Y27632, an inhibitor of rho-dependent kinase, but not when these cells were treated with ML-7, an inhibitor of myosin light chain kinase. Our results provide insight into the mechanobiology of PDL cells, which may contribute towards the development of therapeutic strategies for periodontal healing and tissue regeneration.

## Introduction

The periodontal ligament (PDL) connects the tooth to the alveolar bone and transmits occlusal forces to the periodontium, both of which are critical for mastication. Damage to the PDL, either through disease or injury may impair mastication and/or lead to tooth loss. Clinical therapies for periodontal regeneration, such as open flap debridement, guided tissue regeneration (GTR), or tissue-engineering approaches, share the common goal of harnessing the native process of wound healing in order to restore the PDL to its original form and function [1–3]. After the inflammation phase, new PDL cells, namely stem cells and fibroblasts, migrate into the wound site and rebuild the tissue [4]. PDL cells are critical to wound healing, for they synthesize new collagen fibers and align the fibers by their contractile activity [5,6]. As a result, both cellular contractility and migration are key components in PDL regeneration, so mechanisms that regulate their activity may provide new clinical strategies.

Hyaluronan (HA) is a nonsulfated, linear glycosaminoglycan found abundantly in the PDL [7]. HA has been linked to periodontal regeneration by playing an anti-inflammatory role that prevents tissue damage and facilitates healing [8]. In spite of its abundance in many types of malignant tumors and its promotion of tumor progression [9], HA is safe for periodontal treatments. Its application has shown early promise in clinical studies, but with moderate results [10]. HA is thought to be involved in stimulating proliferation, differentiation, contraction, and/or migration in many cell types [11–19]. However, there has been discrepancy in results with HA, which has been attributed to its cell-specific response and the molecular weight of HA [20]. Thus, defining the responses of PDL cells to HA is important to improve the strategies for periodontal regeneration.

PDL cells express CD44 [21,22], which is the principal receptor to HA. CD44 is a single-chain molecule composed of an N-terminal extracellular domain containing the ligand-binding sites, a membrane-proximal region, a transmembrane segment, and a cytoplasmic tail [23]. The molecular size of CD44 ranges from 80 to 150 kDa depending on variable splicing of at least 11 of the 21 exons coding for CD44 and post-translational modifications [24]. In particular, PDL cells express isoforms CD44s and CD44H [21,22]. CD44 expression upregulates proliferation and mineralization in PDL cells [24], but its effects on PDL contractility and migration are less clear.

The binding of HA to CD44 triggers signals proposed to affect cell migration and contractility through RhoA and Rac1 [9,25,26]. In particular, RhoA activates downstream effector rho-dependent kinase (ROCK) [27]. ROCK phosphorylates the regulatory light chain of nonmuscle myosin and prevents myosin phosphatase activity, both of which lead to a greater amount of nonmuscle myosin bipolar filaments. In another pathway, myosin light chain kinase (MLCK) regulates the formation of nonmuscle myosin bipolar filaments by phosphorylating the regulatory light chain of myosin through a Ca^2+^/calmodulin-dependent pathway [28]. Bipolar filaments interact with F-actin, leading to actomyosin-mediated contractility and cell migration [27,29]. Due to the fact that ROCK and MLCK have distinct roles in contractility and migration [30], it is plausible that HA-CD44 interactions in conjunction with the ROCK pathway mediate PDL cell contractility and migration; however, this relationship has yet to be established.

The objective of this study is to define the effects and mechanism of action of exogenous HA on PDL cells such that an understanding of HA-CD44 interactions may be applied to the development of clinical techniques for periodontal regeneration. Here, we hypothesize that HA-CD44 interactions mediate contractility and migration in PDL cells. As a first step, we examine the effect of exogenous HA on contractility and migration of human PDL cells. Our results demonstrate that cells supplemented with exogenous HA appear more contractile and less migratory. To further establish the role that HA plays on PDL cells, CD44 knockout (KO) mice are used as an *in vitro* model. Herein, we find that CD44-KO PDL cells appear more migratory and less contractile, even following exogenous stimulation with HA when compared to wild-type (WT) cells. Finally, HA-CD44 interactions are abrogated when PDL cells are treated with a ROCK inhibitor, Y27632, but not when treated with ML-7, an inhibitor of MLCK.

## Results

### Exogenous HA increases contractility and reduces migration in human PDL cells

**Figure 1.**
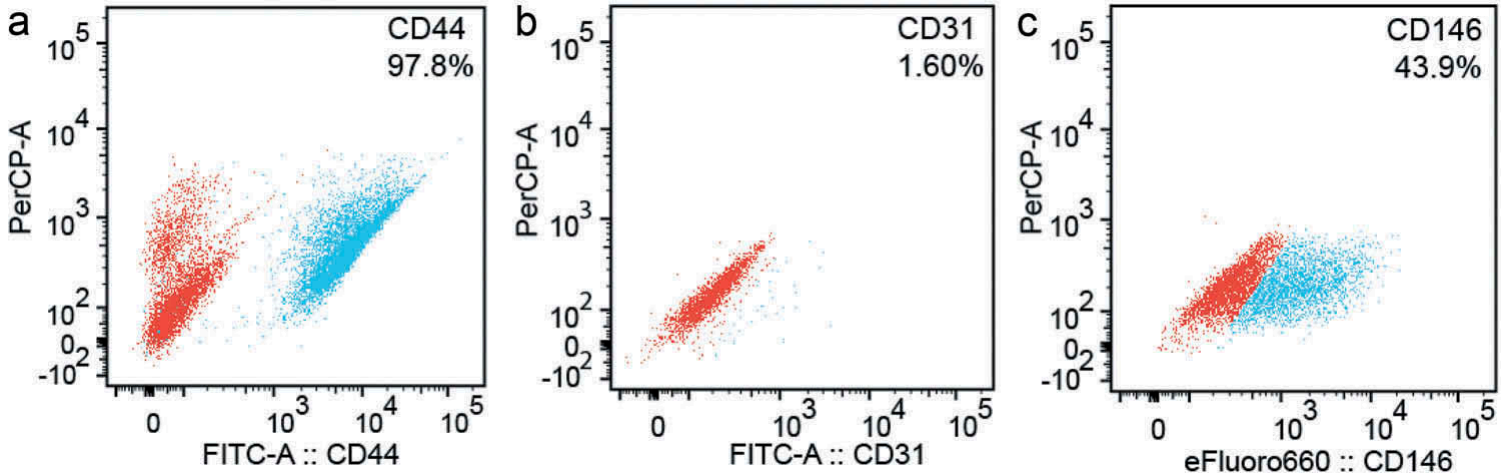
**Characterization of human PDL cells using flow cytometry**. The data shows that (a) 97.8% of human PDL cells expressed the CD44 receptor, (b) 1.60% of the cells expressed the CD31 receptor (endothelial cell line marker) and (c) 43.9% of the population expressed the CD146 receptor (stem cell marker). Red is the untagged control cell population and blue is the cell population tagged for CD44, CD31 or CD146.

The overall expression of the CD44 receptor in human PDL cells was characterized using flow cytometry (Figure 1(a)) and the data showed that 97.8% of the cells expressed this receptor. Furthermore, we found that 1.60% of the cells in the population were positive for CD31 (Figure 1(b)), an endothelial cell marker, and 43.9% were positive for CD146 (Figure 1(c)), a stem cell marker. In addition, human PDL cells cultured *in vitro* showed a spindle-shaped, fibroblast-like phenotype. These findings indicate that PDL cells were comprised largely of fibroblasts and some expressed stem cell markers. Moreover, the CD44 receptor is present in almost the entire population.

**Figure 2.**
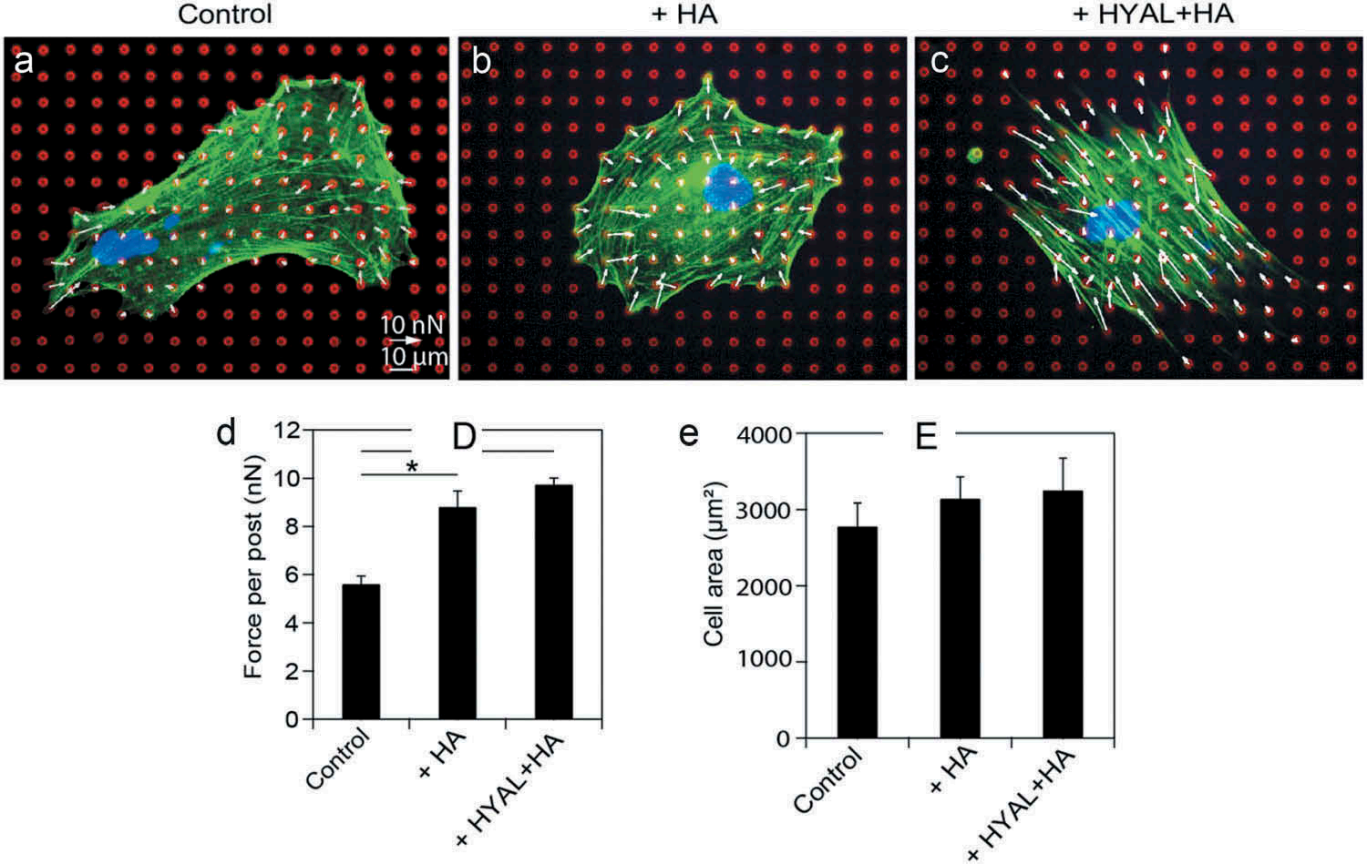
**Exogenous HA increased contractility in serum-starved human PDL cells**. Representative fluorescent images and traction forces of a fixed and stained (a) human PDL cell, (b) PDL cell treated with hyaluronan (HA), and (c) and PDL cell treated with hyaluronidase (HYAL) and HA, (red: microposts; green: actin; blue: nucleus). Traction forces were measured by analyzing the deflections of the microposts and reported as force vectors (arrows). (d) Traction forces of human PDL cells exposed to exogenous HA (population size of experimental repetition: n_1_ = 11, n_2_ = 18, n_3_ = 20; *P* = 0.011) or HYAL+HA (n_1_ = 10, n_2_ = 13, n_3_ = 13; *P* = 5.58e-4) indicate that both treatments induced significant cell contractility compared to the controls (n_1_ = 10, n_2_ = 17, n_3_ = 16). (E) Cell spread area remained comparable to controls (n_1_ = 10, n_2_ = 17, n_3_ = 16), such that it was unaffected by HA (n_1_ = 11, n_2_ = 18, n_3_ = 20) or the combination of HYAL+HA (n_1_ = 10, n_2_ = 13, n_3_ = 13) in human PDL cells. Data shown as average ± SEM from three experimental replicates. Asterisk indicates *P* < 0.05 (Student t-test).

To examine changes in contractility and migration in response to exogenous, low molecular weight HA, we seeded human PDL cells onto arrays of PDMS microposts or onto glass-bottom dishes coated with PDMS. The surface of the PDMS of the microposts and glass-bottom dishes were coated with plasma-derived fibronectin to promote cell attachment. PDL cells appeared to grow normally on the microposts, displaying similar morphological features to cells grown on culture dishes. In order to limit any exogenous HA, hyaluronidase (HYAL) was applied to human PDL cells for 1 hour prior to treating with HA. In comparison to the controls (Figure 2(a)), we observed an increase in stress fibers in these cells in response to either exogenous HA (Figure 2(b)) or a sequential combination of exogenous HYAL and HA (Figure 2(c)). Next, we examined whether exogenous HA affected contractility, and measured the traction forces of PDL cells by analyzing the deflection of the microposts. In comparison to PDL controls, we observed an increase in traction forces in response to either exogenous HA or a sequential combination of exogenous HYAL and HA (Figure 2(d)). Furthermore, to determine if the spreading of human PDL cells was affected by HA or a sequential exposure to HYAL and HA, we analyzed the spread area of the cells. We found that the cell area of human PDL cells remained unaffected by HA or the combination of HYAL and HA (Figure 2(e)). Further analysis was done to rule out the effect of donor variability on traction forces (Fig. S1A, B). In our pilot studies, we treated human PDL cells with and without HYAL and found that their immunofluorescent staining for HA had intensities that were similar for both conditions (Fig. S2A-C). Moreover, HYAL-treated cells had similar morphology and spread area as controls (Fig. S2D). Taken together, we conclude that the effect of HYAL treatment was minimal in this study.

**Figure 3.**
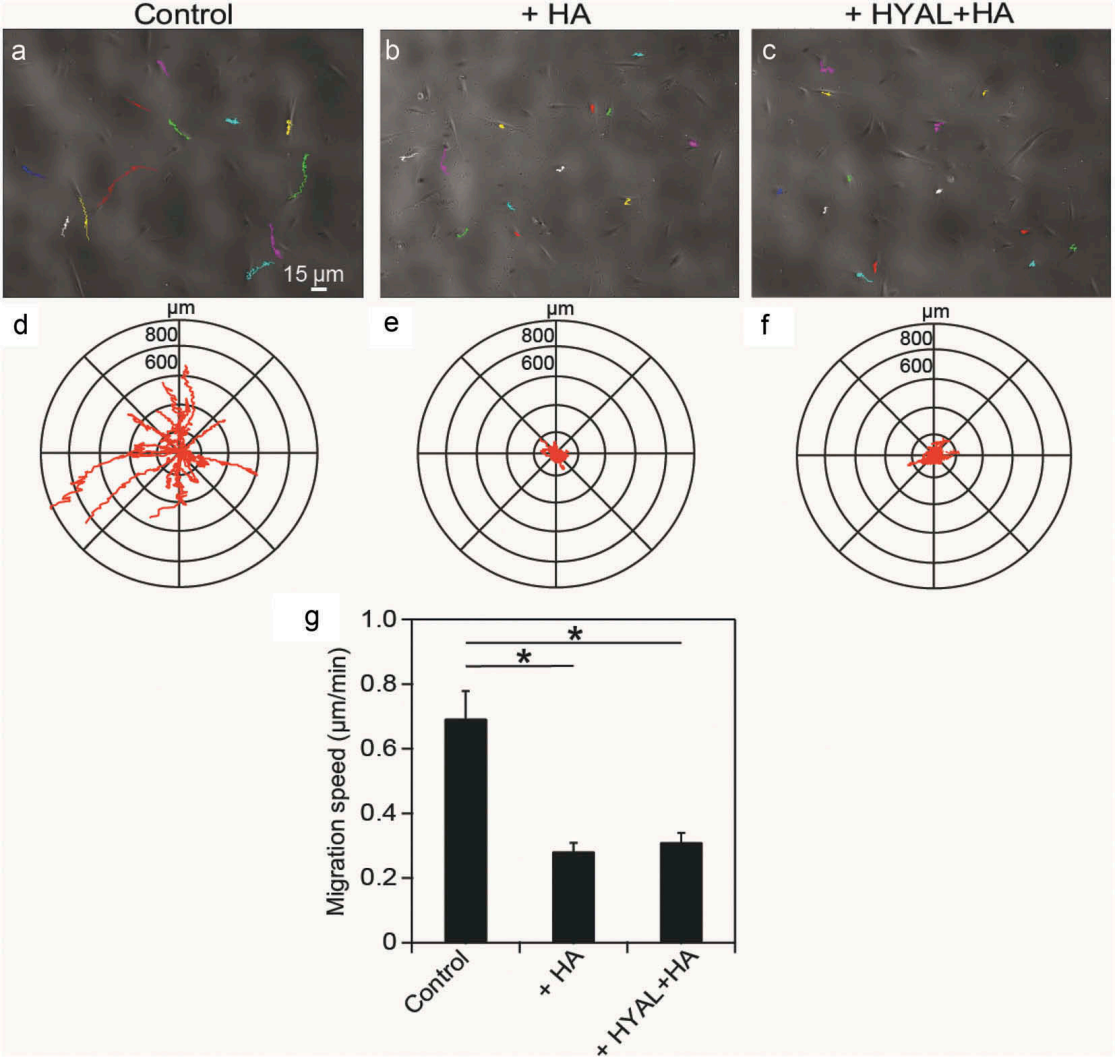
**Exogenous HA reduced migration speeds in human PDL cells**. Time-lapse phase contrast movies were obtained to observe the migration of (a) control, (b) HA-treated and (c) HYAL+HA-treated human PDL cells. Representative phase contrast images were overlaid with the migration tracking data, where each color trace represents the path of a cell. Collecting the migration data for (d) control (population size of experimental repetition: n_1_ = 6, n_2_ = 5, n_3_ = 5), (e) HA-treated (n_1_ = 6, n_2_ = 4, n_3_ = 5), and (f) HYAL+HA-treated PDL cells (n_1_ = 6, n_2_ = 5, n_3_ = 6) revealed that the controls explored a wider territory than either HA-treated or HYAL+HA-treated PDL cells. (g) Migration speed was considerably reduced for HA-treated (*P* = 9.57e-3) and HYAL+HA-treated (*P* = 0.013) PDL cells as compared to controls. Data shown as average ± SEM from three experimental replicates. Asterisk indicates *P* < 0.05 (Student t-test).

HA-CD44 interactions can affect cell migration so we assessed the migration behavior of serum-starved human PDL cells in response to exogenous HA. We used a cell-tracking assay with time-lapse microscopy images taken at 5 min intervals over a 15 hour period in order to trace the migration paths of human PDL cells (Figure 3(a-c)). The phase contrast images overlaid with the migration tracks shown in Figure 3(a-c) are representative of the cell populations examined. The experiments indicated that human PDL cells migrate less when treated with either exogenous HA or a sequential combination of exogenous HYAL and HA. Collecting the migration tracks for all cells analyzed on a rose plot, we observed that over a 15 hour period, human PDL cells migrated shorter distances from their starting points in response to either exogenous HA (Figure 3(e)) or a sequential combination of exogenous HYAL and HA (Figure 3(f)) as compared to PDL controls (Figure 3(d)). Moreover, these cells had lower migration speeds on average as compared to the controls (Figure 3(g)). Taken together, our results suggest strong evidence that HA affects contractility in human PDL cells, which in turn causes these cells to move slower and migrate shorter distances.

### CD44 KO cells migrated faster despite reduced contractility

**Figure 4.**
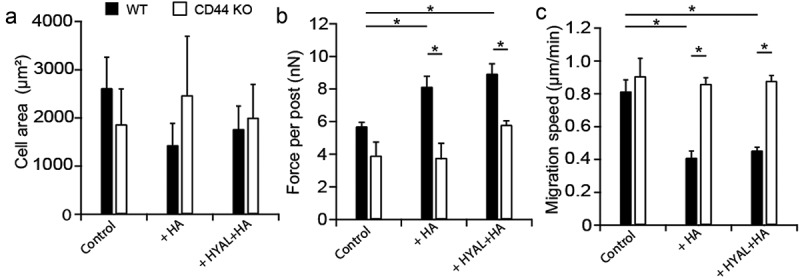
**CD44 KO cells migrated faster despite reduced contractility**. (a) Cell spread area remained unaffected as a result of exogenous HA or a sequential exposure to HYAL+HA in both serum-starved WT (black bars) and CD44 KO (white bars) fibroblast areas. (b) For PDL WT cells, traction forces significantly increased in the presence of both exogenous HA (*P* = 0.026) or the combination of HYAL+HA (*P* = 0.026) compared to the untreated WT. However, CD44 KO cells were unaffected by the presence of exogenous HA in either conditions. Finally, no statistical difference was observed in traction force between CD44 KO and WT cells. However, both CD44 KO cells treated with exogenous HA (*P* = 0.025) and the sequential combination of HYAL+HA (*P* = 8.03e-3) appeared significantly less contractile than their WT counterparts. (c) For migration, PDL WT cells in the presence of exogenous HA (*P* = 0.026) or the sequential combination of HYAL+HA (*P* = 0.029) appeared less migratory than the WT control cells. However, CD44 KO cells appeared significantly more migratory regardless of exogenous stimulation with HA or HYAL+HA. Furthermore, when comparing CD44 KO to WT cells, we found no statistical difference in migration speed between CD44 KO cells compared to WT. However, CD44 KO cells treated with exogenous HA (*P* = 0.022) or the sequential combination of HYAL+HA (*P* = 0.010) appeared more migratory than their WT counterparts. Data shown as average ± SEM from three experimental replicates (Table S1). Asterisk indicates *P* < 0.05 (one-way ANOVA with a Bonferroni’s and Fischer post-hoc adjustment).

To further investigate the role of HA in mediating contractility and migration via its interaction with the CD44 receptor, we considered a CD44 KO mouse model. Similarly to human PDL cells, CD44 KO and WT PDL cells showed a spindle-shaped, fibroblast-like morphology when cultured *in vitro*. Here, WT and CD44 KO PDL cells were cultured on arrays of microposts or glass-bottom dishes as aforementioned. We found that cell area remained unaffected as a result of exogenous HA or a sequential exposure to HYAL and HA (Figure 4(a)). Next, we examined the effect of HA on traction forces in CD44 KO and WT cells. We observed an increase in the traction forces of WT cells in the presence of both exogenous HA or the combination of HYAL and HA (Figure 4(b)). Conversely, the traction forces of CD44 KO cells were unaffected by the presence of exogenous HA in either conditions (Figure 4(b)). Moreover, when statistically comparing CD44 KO cells to WT cells, we found no statistical difference in traction force between CD44 KO cells compared to WT cells. However, CD44 KO cells treated with exogenous HA and the sequential combination of HYAL and HA appeared significantly less contractile than their WT counterparts (Figure 4(b)).

Finally, we investigated the role of exogenous HA in mediating changes in migration. We found that in the presence of exogenous HA or the sequential combination of HYAL and HA, WT cells appeared less migratory than CD44 KO controls (Figure 4(c)). However, CD44 KO cells appeared significantly more migratory with exogenous HA or HYAL and HA (Figure 4(c)). Furthermore, when comparing CD44 KO to WT cells, we found no statistical difference in migration speed between CD44 KO cells compared to WT cells. In addition, CD44 KO cells treated with exogenous HA or the sequential combination of HYAL and HA appeared more migratory than their WT counterparts (Figure 4(c)). In light of these findings, the results indicate that CD44 plays a role in the contractility of PDL cells in response to HA. Furthermore, the absence of the CD44 receptor causes CD44 KO cells to move faster regardless of the presence of exogenous HA.

### HA-CD44 interaction mediated contractility through ROCK

**Figure 5.**
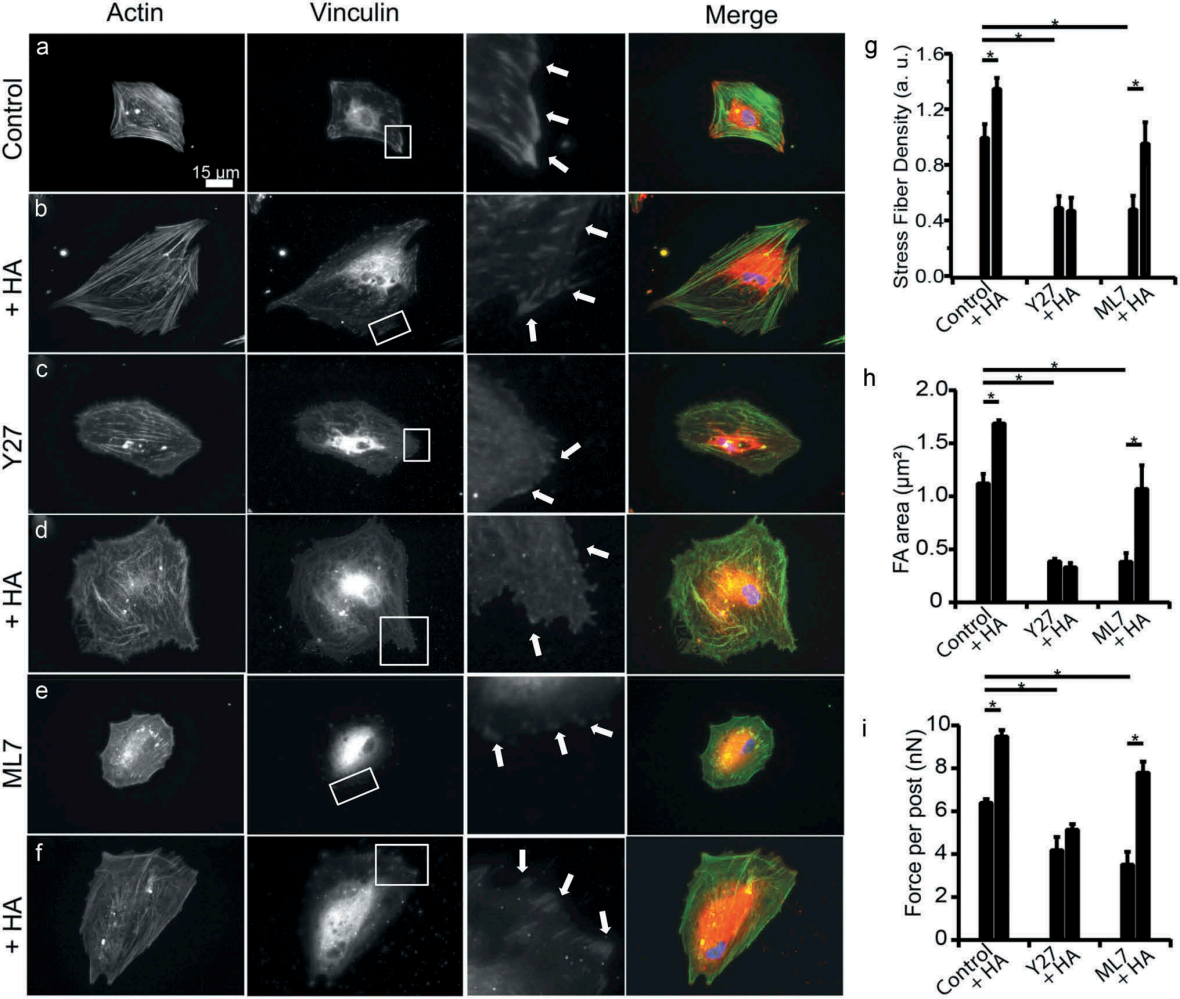
**HA-CD44 interaction mediated contractility through ROCK pathway**. Human PDL cells were cultured on PDMS-coated glass coverslips. Representative fluorescent images of a fixed and stained (a) control, (b) HA-treated, (c) Y27-treated, (d) Y27+ HA-treated, (e) ML7-treated, (f) ML7+ HA-treated human PDL cells (red: vinculin; green: actin; blue: nucleus). (g) Y27 (*P* = 2.27e-3) and ML7 (*P* = 3.57e-3) treatments caused a reduction in stress fiber density relative to control PDL cells; however, treating the cells with exogenous HA caused an increase in stress fiber density for controls (*P* = 1.72e-3) and ML7-treated PDL cells (*P* = 0.019), but not in Y27-treated cells. (h) Regarding the focal adhesion assays, Y27 (population size of experimental repetition: n_1_ = 7, n_2_ = 5, n_3_ = 13; *P* = 9.63e-4) and ML7 (n_1_ = 9, n_2_ = 8, n_3_ = 11; *P* = 2.67e-3) treatments caused focal adhesions to decrease in size relative to control human PDL cells (n_1_ = 15, n_2_ = 13, n_3_ = 14); however, treating the cells with exogenous HA caused an increase in focal adhesion size for controls (n_1_ = 29, n_2_ = 20, n_3_ = 17; *P* = 2.71e-3) and ML7-treated PDL cells (n_1_ = 14, n_2_ = 11, n_3_ = 8; *P* = 0.039), but not Y27-treated cells (n_1_ = 7, n_2_ = 6, n_3_ = 10). (i) After stimulating with HA (n_1_ = 5, n_2_ = 12, n_3_ = 2; *P* = 4.44e-4), we found a significant increase in traction forces compared to untreated controls (n_1_ = 7, n_2_ = 8, n_3_ = 4). Conversely, we found that after treating cells with Y27 (n_1_ = 6, n_2_ = 11, n_3_ = 4; *P* = 0.020) and ML7 (n_1_ = 9, n_2_ = 3, n_3_ = 7; *P* = 7.66e-3), we found a significant loss in traction forces. After adding exogenous HA to Y27-treated (n_1_ = 6, n_2_ = 8, n_3_ = 7) or ML7-treated (n_1_ = 9, n_2_ = 7, n_3_ = 7) PDL cells, we observed a significant increase in traction force only in ML7-treated cells (*P* = 0.028) and not Y27-treated cells. Data shown as average ± SEM from three experimental replicates. Asterisk indicates *P* < 0.05 (one-way ANOVA with a Bonferroni’s and Fischer post-hoc adjustment).

ROCK and MLCK play a major role in governing the cytoskeleton, focal adhesions, and actin-myosin contractility, but only ROCK has been seen to be affected by HA binding to CD44. Here, we investigated whether ROCK signaling could be involved in the increase in traction forces and reduction in cell migration caused by HA-CD44 interactions. ROCK was inhibited in serum-starved human PDL cells with Y27 and MLCK was inhibited with ML7. PDL cells treated with Y27 or ML7 had disrupted morphologies and reduced organization of their actin cytoskeletons as compared to control PDL cells (Figure 5(c,e,g)). Furthermore, stress fiber density for both controls and ML7-treated PDL cells significantly increased in the presence of HA (Figure 5(b,f,g)); however, this was not observed in Y27-treated cells (Figure 5(d,g)). The focal adhesions of PDL cells treated with Y27 or ML7 were not well-defined, punctate structures (Figure 5(c,e,h)). Furthermore, we observed no change in the morphology or size of focal adhesions for Y27 treated cells after the addition of exogenous HA (Figure 5(d,h)). Conversely, ML7-treated and control cells showed a significant increase in focal adhesion size with exogenous HA (Figure 5(f,h)).

Inhibition of ROCK and MLCK are well known to decrease traction forces compared to uninhibited cells [31–34]. After stimulating uninhibited control cells with HA, we observed a significant increase in traction forces (Figure 5(i)). Conversely, we found that treating PDL cells with Y27 and ML7 led to a significant loss in traction forces. After stimulating Y27- and ML7-treated cells with HA, we observed a significant increase in traction force in ML7-treated cells and Y27-treated cells remained unchanged (Figure 5(i)). Both ROCK and MLCK can regulate traction forces, but our results indicate that inhibiting MLCK alone is not sufficient to abrogate the response in traction forces due to exogenous HA. Therefore, these findings suggest that HA-CD44 interactions in human PDL cells mediate changes in their contractility via the ROCK pathway independent of the MLCK pathway.

## Discussion

Earlier studies have found that HA affects healing at different stages during wound repair: inflammation, cell migration, proliferation, angiogenesis, and matrix remodeling [35–40]. Our results provide a new context for HA-CD44 interactions that highlight the mechanobiology of PDL cells. In particular, our findings demonstrate that PDL cells produce higher traction forces when in the presence of exogenous HA. These cells move considerably slower, exhibiting shorter migration tracks, and thus having reduced migration speeds. In contrast, CD44 KO PDL cells exhibit higher migration speeds and less contractile forces in the presence of exogenous HA than their WT counterparts, suggesting that contractility and migration in the PDL cells may be regulated through the interaction of HA and CD44.

Based on previous studies, the effect of HA on cellular contractility and migration appears to be cell-type dependent. Both primate aortic smooth muscle cells and adventitial fibroblasts significantly increased matrix contractions on collagen I gels containing HA [14]. Furthermore, the addition of HA has been found to reduce migration, proliferation, and cell adhesion in CHO cells [12]. Consistent with these findings, we found that PDL cells appear to move significantly slower with increased cytoskeletal tension. In contrast, other studies have shown that the addition of exogenous HA significantly enhanced migration in mesenchymal stem cells [41] and in human corneal epithelial cells [19]. These studies indicate that cells are likely to possess a response to HA in a manner that is cell-type specific. A previous study with PDL cells observed increased migration of the cells through Boyden chambers with membranes coated with HA and in the presence of FGF-2 [16]. However, we note distinct differences in the experimental approaches of this work and our own: our experiments were performed with soluble HA on a fibronectin-coated surface and without a chemotactic stimulus.

Cell attachment and spreading were previously shown to be unaffected by exogenous HA in skin fibroblasts [42]. However, it has also been shown that exogenous HA can promote lamellipodial formation in smooth muscle cells through the interaction of HA with the receptor for HA-mediated motility (RHAMM) [43]. Here, we found that exogenous HA did not influence cell spreading, which is more reminiscent of fibroblast behavior. Thus, spreading due to exogenous HA, like contractility and migration, may be a specific response due to cell-type.

PDL cells are thought to be mechanosensitive, incorporating physical cues arising in their tissue microenvironment in order to carry out their proper physiological function [5,24,44,45]. Recent work has shown that lack of CD44 in KO mice may promote greater tooth extrusion at early functional stages of tooth eruption [44]. Since HA binding to CD44 has been shown to regulate focal adhesion dynamics involved in cell-cell and cell-matrix interactions [46], the absence of HA-CD44 interactions could generate defects in the connections between cellular and extracellular matrix components of the PDL. Here, we observed that the traction forces of CD44 KO cells were insensitive to exogenous HA. This finding indicates that the response of PDL cells to their microenvironment is impaired in CD44 KO cells, and may compromise the ability of the ligament to stabilize the tooth against occlusal forces.

ROCK and MLCK are well known to regulate both traction forces and focal adhesion formation [31,47]. Although the exact mechanism, or the role of exogenous HA on human PDL cell contractility is not entirely understood, we hypothesize that there are two pathways that govern PDL cell contractility through HA-CD44 interactions. The first is through the activation of ROCK and the second is through MLCK. The binding of HA to CD44 triggers the signaling of RhoA through the leukemia-associated Rho guanine nucleotide exchange factor (LARG) [9]. RhoA participates in the regulation of various cellular functions including stress fiber and focal adhesion formation through the activation of ROCK [48]. ROCK phosphorylates the regulatory light chain of nonmuscle myosin (MLC) and inhibits myosin phosphatase (MLCPase), both of which lead to increased actomyosin contractility. The second plausible mechanism through which exogenous HA may affect PDL cell contractility is through the MLCK pathway. When HA binds to CD44, it can induce CD44–ankyrin interactions that cause Ca^2+^ mobilization through the IP_3_ receptor (IP_3_ R), leading to the activation of MLCK through a Ca^2+^/calmodulin-dependent pathway [9,49,50]. Like ROCK, MLCK directly phosphorylates MLC, leading to increased actomyosin contractility [27]. We observed that ROCK-inhibited cells and MLCK-inhibited cells showed significantly reduced forces and focal adhesion areas compared to the untreated controls (Figure 5(h,i)). These results indicate that activation of ROCK and MLCK are important in PDL cell contractility. However, MLCK-inhibited cells showed a response to the presence of exogenous HA by a significant increase in force and focal adhesion area (Figure 5(h,i)). On the other hand, ROCK-inhibited cells showed no response in force and focal adhesion area in the presence of HA. These results suggest that the increase in PDL cell contractility and focal adhesion formation in response to HA-CD44 interactions in human PDL cells are likely acting through the ROCK pathway in a manner that is independent of MLCK. However, future studies are required to provide a full and complete understanding of the role of HA in regulating human PDL cell contractility.

The results of our study provide insight into the potential effects of HA-CD44 interactions on PDL cells during regenerative treatments. In our investigation, PDL cells appeared more contractile and less migratory in the presence of HA, suggesting that these cells could also have strong attachment to fibronectin in the granulation tissue [51] or exposed Sharpey’s fibers on the root surface [1]. Establishing a PDL cell population in a periodontal defect is an important requirement for the remodeling and rebuilding of the periodontium. Previous studies have demonstrated that exogenous application of HA significantly enhanced the remodeling of the extracellular matrix, which showed an increase in the organization of the collagen fibers [52,53]. The rebuilding activities of fibroblasts have been observed in collagen gels in which fibroblasts rearrange and consolidate collagen fibrils into fiber bundles, as well as exert cytoskeletal forces on individual collagen fibers [54,55]. During development of periodontal fiber bundles, PDL cells form cellular contacts that define a sheath-like space within which collagen fibrils are secreted and aligned [56,57]. The performance of PDL cells in such modeling activities is likely to depend on traction forces exerted through cell-cell and cell-matrix interactions, and such cytoskeletal forces may in turn be mediated by HA-CD44 interactions. If so, high PDL cell traction forces may be a necessary component to the assembly of individual collagen fibers into the principal fiber bundles during PDL regeneration.

The mechanisms by which HA-CD44 interactions promote the healing responses of PDL cells are not completely understood, but are a promising area for future investigations. Clinical studies implicate HA as a useful supplement for effective periodontal healing. The efficacy of HA was evaluated in the treatment of periodontal defects and showed significant reduction in pocket depth and promotion of attachment [58]. Another study in periodontal defects that investigated the function of a biological hydrogel of recombinant FGF-2 in a HA carrier showed significant improvement in healing after one year of treatment [59]. In addition, application of HA gel (Gengigel®) was shown to be an antimicrobial agent and a means to enhance wound healing [10]. Further *in vitro* and *in vivo* study of HA-CD44 interactions at specific stages of periodontal regeneration could advance the development of therapeutic strategies for healing periodontal defects. Our results provide evidence that HA-CD44 interactions are associated with PDL cell functions pertaining to wound healing, and specifically affect mechanotransduction mechanisms associated with traction forces and migration. Overall, these results may further our understanding of PDL mechanobiology and provide a rationale for future more detailed and clinically relevant experimentation that may advance therapeutic strategies for healing and tissue regeneration.

## Materials and methods

### Cell culture

Primary human PDL cells were isolated from healthy third molars extracted from three de-identified donors in the course of orthodontic treatment from the ages of 18–32. Informed consent from donors was obtained in compliance with the Institutional Review Board of the University of Washington. PDL cell cultures were established from scrapings from the middle third of the roots. The fibroblast-like nature of these human PDL cell populations was defined in a previous study using immunohistochemical staining for vimentin, alpha smooth muscle actin, epithelial keratin, and neurofilament protein (Santa Cruz Biotechnology, Santa Cruz, CA, USA). Additionally, PDL cells showed higher staining for alkaline phosphatase than gingival fibroblasts [60]. Human PDL cells were cultured in Dulbecco’s modified Eagle’s medium (DMEM) (Gibco Life Technologies, Grand Island, NY, USA) with 10% fetal bovine serum (Hyclone Laboratories, Logan, UT, USA) and 1% streptomycin/penicillin (Hyclone Laboratories, Logan, UT, USA) and maintained in an incubator at 37°C and 5% CO_2_ incubator. The passage numbers for human PDL samples ranged from five to seven.

CD44 KO mice were obtained from the laboratory of Dr. Tak Mak from the University of Toronto and WT mice (strain C57BL/6) were obtained from Jackson Laboratories (Bar Harbor, ME, USA). The CD44 KO mouse model was created through disruption of expression of exons encoding the invariant N-terminus region of the molecule; therefore, preventing expression of all known isoforms of CD44 [61]. The breeding and harvesting of dental tissue from CD44 KO and WT mice was performed under protocols approved by the IACUC at the University of Washington. Both male and female mice were culled by cervical dislocation between 23–30 days postnatal and maxillary and mandibular molars were extracted. Primary mouse PDL cell cultures were digested from the tooth surface in a solution of 3 mg/ml collagenase type I and 4 mg/ml dispase (Gibco Life Technologies, Grand Island, NY, USA) at 37°C for 30 min. The digested solution was passed through a 70 μm nylon mesh strainer (Fisher Scientific, Fair Lawn, NJ, USA) and the cell suspension was pelleted in a desktop centrifuge at 1000 × g for 5 min. Cells were resuspended and grown in DMEM (Gibco Life Technologies, Grand Island, NY, USA) with 20% fetal bovine serum (Hyclone Laboratories, Logan, UT, USA) and 1% streptomycin/penicillin (Hyclone Laboratories, Logan, UT, USA).

### Flow cytometry

Human PDL cells were grown in two T25 dishes (Greiner Bio One, Monroe, NC, USA) to confluency. The cells were then trypsinized and resuspended in flow cytometry buffer (eBioscience, San Diego, CA, USA). These cells were subsequently blocked for non-specific interactions using human Fc receptor binding inhibitor (eBioscience, San Diego, CA, USA) at room temperature. For surface staining we used the following antibodies: rat mAb CD44-FITC (1:50 dilution, ab19622, Abcam, Cambridge, MA, USA), anti-CD146 (1:40 dilution, 50–1469, eBioscience, San Diego, CA, USA) and anti-human CD31 (1:40 dilution, 11–0319, eBioscience, San Diego, CA, USA), and incubated for 30 min in the dark at 2–8°C. The cells were washed three times with flow cytometry buffer after centrifugation at 400–600xg for 5 min. After the final wash, the cells were suspended in flow cytometry buffer and 7-AAD staining (eBioscience, San Diego, CA, USA), before being fixed with intracellular fixation buffer (eBioscience, San Diego, CA, USA). The tubes were then analyzed using a LSRII cytometer equipped with 488 nm, 561 nm, 640 nm, 405 nm and 350 nm lasers and all flow cytometry data were analyzed with Flow Jo software. Side and forward scatter of aggregates in cell lysates were determined using log scale plots. Voltage settings for the PerCp-A, FITC and efluoro660 channels were kept constant for all experiments described.

### Micropost preparation, staining, imaging and force measurements

Arrays of microposts were made from a 10:1 ratio of polydimethylsiloxane (PDMS) (Dow-Corning, Midland, MI, USA) and cured on top of glass coverslips for 6 hours at 110°C via a replica-molding process as previously described [62]. Each micropost in the array had a height of 8 µm, diameter of 2.1 µm, and center-to-center spacing of 9 µm. The tops of the microposts were coated with plasma-derived fibronectin (50 μg/mL, Corning, Pittsburgh, PA, USA) by microcontact printing [62]. After stamping, the arrays were immersed in 2 μg/mL bovine albumin serum conjugated Alexa Fluor 594 (Molecular Probes, Grand Island, NY, USA) for 1 hour to stain the PDMS so that the microposts could be observed with fluorescent microscopy. Next, the arrays were submerged in 0.2% Pluronic F-127 (Sigma-Aldrich, St. Louis, MO, USA) for 30 min to block the adsorption of additional proteins to the PDMS. Cells were seeded onto the arrays at a density of 10^5^ cells/cm^2^ and allowed to attach for 2 hours. Unattached cells were washed away with additional media.

After 24 hours of culture, the cells were serum-starved in media with 0.1% fetal bovine serum for 4 hours to reduce the production of endogenous HA. Samples were incubated with either fluorescein-tagged HA (Sigma-Aldrich, St. Louis, MO, USA; 100 µg/mL, ~800 kDa) for 1 hour or a combination of hyaluronidase (4 U/mL, Sigma-Aldrich, St. Louis, MO, USA) for 1 hour followed by fluorescein-tagged hyaluronan for 1 hour. Cells on the microposts were fixed with 4% paraformaldehyde and permeabilized with 0.5% Triton X-100. The samples were stained with phalloidin Alexa Fluor 488 (Molecular Probes, Grand Island, NY, USA) and Hoescht 33342 (Molecular Probes, Grand Island, NY, USA). The concentration used for exogenous HA was within the range of concentrations (0.06–1 mg/mL) used in previous studies [42,63,64].

Imaging was carried out on an epi-fluorescent microscope (Nikon TEi) (Nikon, Instruments Inc., Melville, NY, USA) with a 40× oil objective (NA = 1.00). Images obtained from epi-fluorescence microscopy were analyzed with image analysis codes written in MATLAB (The MathWorks, Natick, MA, USA) to measure the deflection of each micropost underneath a cell, as well as the spread area of the cell as previously described [62,65]. Each micropost had a height of 8 µm, diameter of 2.1 µm, and spring constant of k = 16.6 nN/µm, resulting in an effective shear modulus of 1.7 MPa. For the aspect ratio of the micropost (height/diameter = 3.9), a correction factor k_tilt_ = 0.81, was included in the calculation to account for tilting at the compliant base of the microposts [66].

### Migration assay

PDL cells were grown on glass-bottom dishes (MatTek Corp, Ashland, MA, USA) coated with a thin layer of PDMS and fibronectin (50 μg/mL). The cells were imaged every 5 min during a period of 15 hours using time-lapse, phase contrast microscopy on an inverted microscope (Nikon TEi) with a 4× air objective (NA = 0.13) in a 37°C, 5% CO_2_ chamber. An ImageJ plugin (MTrackJ) was used to track the nucleus of the individual cells.

### Focal adhesion and stress fiber quantification

Images obtained from epi-fluorescence microscopy were analyzed in ImageJ. To identify focal adhesions, cells were grown on glass coverslips coated with a thin layer of PDMS and fibronectin (50 μg/mL). The samples were fixed and stained with phalloidin Alexa Fluor 488, Hoescht 33342, mouse anti-vinculin antibody (1:200 dilution, V9131 SIGMA, Sigma-Aldrich, St. Louis, MO, USA), and AlexaFluor 647 conjugated goat anti-mouse IgG antibody (1:200 dilution, A-21235, Molecular Probes, Grand Island, NY, USA). Fluorescent images of focal adhesions were processed by a step-by-step quantitative analysis [67]. In brief, the raw immunofluorescent images were imported into ImageJ and processed by first subtracting the background; second, enhancing the local contrast of the image by using the ImageJ plugin: contrast limited adaptive histogram equalization; third, applying a mathematical exponential in order to further minimize any background noise; fourth, adjusting the brightness and contrast and; finally, running the Laplacian of Gaussian filter [68]. Here, we set both lower and upper bounds on focal adhesion area based on the distribution we observed, which were 0.2 μm^2^ and 4 μm^2^ respectively. To quantify the stress fiber density in the cells, the raw immunofluorescent images of cells stained with phalloidin Alexa Fluor 488 were imported into ImageJ and processed by considering a line profile across the cell [69], which identified stress fibers by their increased fluorescence intensity in relation to areas without stress fibers. The sharp peaks of fluorescence intensity represented individual stress fibers, and the width of the peak indicated the thickness of a stress fiber. The numbers of stress fibers were quantified for each cell and the values presented have been normalized to the control sample.

### Inhibition and stimulation

Human PDL cells were serum-starved in media with 0.1% FBS (Hyclone Laboratories, Logan, UT, USA) for 4 hours prior to adding the treatments. Y-27632 (Y27, EMD Millipore, Billerica, MA, USA) and ML-7 (ML7, Sigma-Aldrich, St. Louis, MO, USA) were stored as stock solutions in DMSO. Inhibition of ROCK or MLCK was achieved by exposing cells to Y27 (20 μM, final concentration) or ML7 (20 μM, final concentration) for 30 minutes, respectively. The concentrations used for Y27 and ML7 have been used in previous studies [70–72].

### Statistical analysis

Data were obtained from three replicates (n_1_, n_2,_ n_3_). All values are presented as the average ± SEM. Samples were analyzed for significance using one-way ANOVA with a Bonferroni’s or Fischer post-hoc adjustments and a Student t-test. Comparisons were considered significant for *P-*values of < 0.05 (marked with asterisks in the figures).
