# Supplementary material for: Hyaluronan-CD44 interactions mediate contractility and migration in periodontal ligament cells
Source: Cell Adh Migr. 2019 Feb 8;13(1):138–50. doi: 10.1080/19336918.2019.1568140 (PMC6527381; doi:10.1080/19336918.2019.1568140)
Supplement: Supplemental Material [file kcam-13-01-1568140-s001.zip › Supplemental Information_revision_Final.docx]

**Supplemental Information**


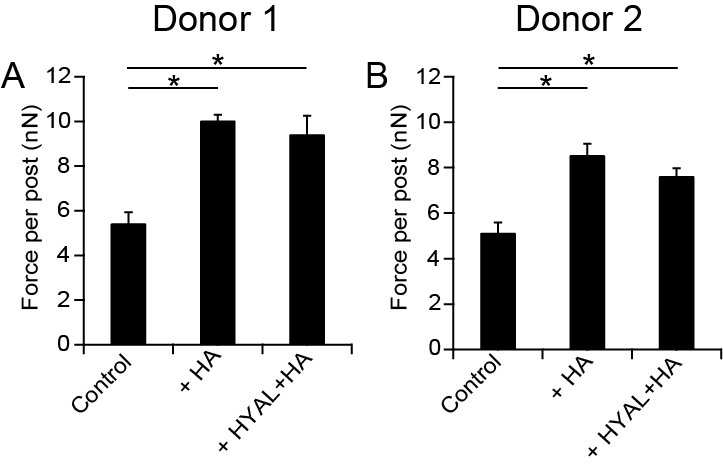


**Fig. S1. Contractility is independent of donor variability**. (A) Traction forces of human PDL cells from Donor 1 exposed to exogenous HA (*P* = 1.22e-3) or HYAL+HA (*P* = 0.014) indicated that they were more contractile than the controls. (B) Traction forces of human PDL cells from Donor 2 exposed to exogenous HA (*P* = 3.11e-3) or HYAL+HA (*P* = 7.29e-4) indicated that they were more contractile than the controls. Data shown as average ± SEM. An asterisk indicates *P* < 0.05.


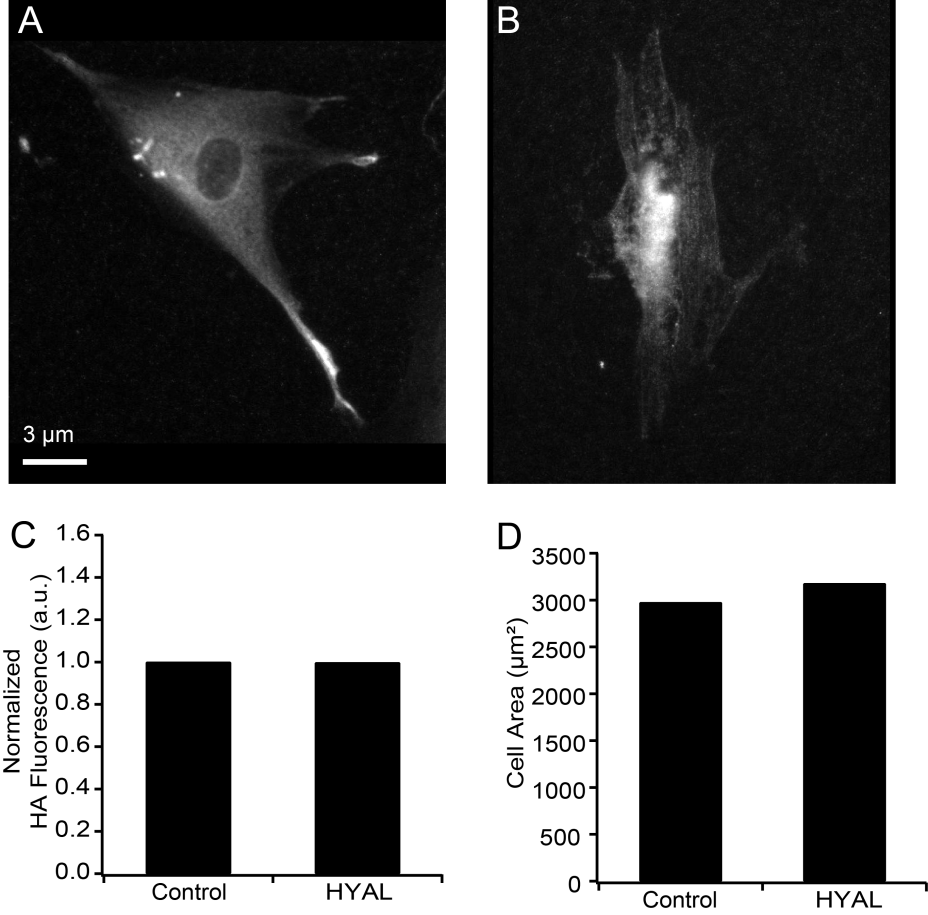


**Fig. S2. Pilot studies of role of HYAL.** Immunofluorescent images of both (A) control and (B) HYAL-treated human PDL cells. In both cases, cells were stained for HA. (C) The normalized HA fluorescence intensity for both control and HYAL-treated cells remained the same. (D) Furthermore, cell spread area also remained comparable between controls and HYAL-treated cells.

10 µm
