# Supplementary material for: Hyaluronan-CD44 interactions mediate contractility and migration in periodontal ligament cells
Source: Cell Adh Migr. 2019 Feb 8;13(1):138–50. doi: 10.1080/19336918.2019.1568140 (PMC6527381; doi:10.1080/19336918.2019.1568140)
Supplement: Supplemental Material [file kcam-13-01-1568140-s001.zip › Table S1.docx]

**Table S1. Sample size for murine PDL cells**.

| Murine PDL cells | Contractility | Migration |
| --- | --- | --- |
| WT | n_1_ = 8 ; n_2_ = 6; n_3_ = NA | n_1_ = 4; n_2_ = 2; n_3_ = NA |
| WT +HA | n_1_ = 11; n_2_ = 9; n_3_ = 11 | n_1_ = 8; n_2_ = 9; n_3_ = 9 |
| WT +HYAL+HA | n_1_ = 16 ; n_2_ = 9; n_3_ = 10 | n_1_ = 6; n_2_ = 9; n_3_ = 9 |
| CD44 KO | n_1_ = 14 ; n_2_ = 6; n_3_ = 9 | n_1_ = 6; n_2_ = 6; n_3_ = 8 |
| CD44 KO +HA | n_1_ = 9; n_2_ = 17; n_3_ = 12 | n_1_ = 5; n_2_ = 7; n_3_ = 6 |
| CD44 KO +HYAL+HA | n_1_ = 10; n_2_ = 15; n_3_ = 10 | n_1_ = 3; n_2_ = 7; n_3_ = 8 |
